# Editorial: New Perspectives in Bereavement and Loss: Complicated and Disenfranchised Grief Along the Life Cycle

**DOI:** 10.3389/fpsyg.2021.691464

**Published:** 2021-05-25

**Authors:** Manuel Fernández-Alcántara, Cyrille Kossigan Kokou-Kpolou, Francisco Cruz-Quintana, María Nieves Pérez-Marfil

**Affiliations:** ^1^Department of Health Psychology, University of Alicante, Alicante, Spain; ^2^School of Psychology, University of Ottawa, Ottawa, ON, Canada; ^3^Mind, Brain, and Behavior Research Center (CIMCYC), University of Granada, Granada, Spain

**Keywords:** complicated grief, disenfranchised grief, bereavement, end of life care, prolonged grief disorder

Losing a loved one is one of the crucial and most difficult experiences that individuals may cope with during their lifetime. Although most people will go through an adaptive grieving process, there is a significant percentage who will encounter difficulties. Recent meta-analytic findings showed that around a 10% in the cases of natural deaths (Lundorff et al., 2017) and a 49% in the cases of unnatural losses (Djelantik et al., 2020) are at high risk of developing complicated (CG) or prolonged grief disorder (PGD). At present, although there are diagnostic criteria in both the next DSM-5-TR and the ICD-11 (Prigerson et al., 2021), there is no consensus on the symptomatology that defines a CG, and there are different proposals in this respect. In addition, it is necessary to conduct research in the areas of identification and clarification of the emotional, cognitive, and behavioral reactions that characterize CG in different populations, such as substance abuse population, people with less economical resources, etc. In this sense, scientific literature has identified different trajectories and types of grief (including anticipatory grief, disenfranchised grief, etc.) where the adaptation to loss can be difficult to manage, especially if the bereavement process is not acknowledged by the social environment. In the latter cases, usually defined as disenfranchised grief, there are multiple examples that may include cases of perinatal grief, loss due to the diagnoses of a chronic condition in oneself or in a close person, grief due to mental illness or in cases of the diagnosis of a disability, loss of a job, loss of a pet or even grief related to the multiple changes in the environment (Setubal et al., 2020; van Eersel et al., 2020; Comtesse et al., 2021).

In the current Research Topic, a series of investigations have been presented to deepen the experience of grief in specific populations using novel methodologies and data analysis. Glickman has shown how the prevalence of PGD in university students (13.4%) is almost similar to that found in the adult population in previous research (Lundorff et al., 2017). The author found that bereavement-related factors, race, history of anxiety or depression, trauma other than the death, or insecure attachment style were associated with increased odds for PGD. Another study (Li et al.), using latent-class analysis, explored the different subgroups of bereaved adults regarding post-traumatic growth (PTG). PTG is related to the positive changes that can be associated with a potentially traumatic experience (Calhoun et al., 2010). The results indicate the complexity of the emotions after the loss of a loved one, and how PTG is linked with depression and anxiety. Two of the studies have also shown the particularities of the bereavement experience in people who are regular substance users. In the first of these, using a network analysis approach Masferrer et al. found that complicated grief was largely independent of patterns of personality disorders, assessed through the Millon Clinical Multiaxial Inventory (with the exception of depressive and paranoid subscales). The second study found that CG patients diagnosed with substance use disorder endorsed more dysfunctional coping responses (e.g., social withdrawal, self-criticism) in comparison with those without CG (Caparrós and Masferrer). These studies suggest the importance of measuring CG symptoms when designing specific psychological interventions in substance abusers.

One of the crucial challenges of current research in the field of grief and bereavement is the identification of those at risk of developing CG or PGD. For that purpose, the development and validation of psychometric instruments to assess grief intensity and intervening variables is mandatory. The identification of people at risk of developing CG or PGD is one of the crucial challenges in the area (Treml et al., 2020). Based on the cognitive behavior model (Boelen et al., 2006; Doering et al.), present the psychometric properties of the German adaptation of the Grief Cognitions Questionnaire. They included the data of the full version of the instrument as well as a reduced version, which can be easily applied in clinical and research contexts.

Finally, three studies have been included regarding interventions and actions directed to the people suffering the loss from different perspectives. In the context of an European Project, Orkibi et al. conducted a mixed-methods study in Master's students to explore what characteristics and attitudes were associated to their interest in being involved, as health care professionals, in palliative and end-of-life care. Variables such as previous care experience and previous loss were identified, in line with previous studies in other samples such as nursing students (Martí-García et al., 2020). In the scoping review conducted by Wojtkowiak et al., the role of rituals and symbolic elements in the grief-related interventions is highlighted. This is a central topic, especially regarding the COVID-19 situation in which we are immersed at the moment and that implies the absence of some of the traditional rituals as well as the social isolation of the bereaved (Kokou-Kpolou et al., 2020). Finally, the effectivity of a novel intervention program based on Katherine Shear's model was presented (Shear et al., 2005). The ten-session program for anticipatory grief in caregivers of dementia patients included modules of psychoeducation, exposure techniques, and role-playing situations to address the specific grief related symptoms. Results showed positive results in improving grief symptoms, caregiver burden, resilience, PTG, and quality of life of family caregivers (Bravo-Benítez et al.).

In sum, this Research Topic adds new evidence about: (1) the profile of CG and the main coping strategies in substance abusers of populations; (2) the effectiveness of interventions for improving ambiguous grief symptomatology; (3) the development and validation of assessment tools that can be used as part of the initial diagnosis or as an outcome of treatment progress and (4) the importance of prevention and social support strategies for both health care professionals that participate in end-of-life care and those suffering from complicated grief and (5) the importance of considering the cultural aspects in the ritual and symbolic interventions in the cases of CG.

## Author Contributions

MF-A, CK-K, FC-Q, and MP-M wrote the first draft of the manuscript. All authors contributed to manuscript revision, read, and approved the submitted version.

## Conflict of Interest

The authors declare that the research was conducted in the absence of any commercial or financial relationships that could be construed as a potential conflict of interest.
